# Socioeconomic Classes among Oldest-Old Women in South Korea: A Latent Class Analysis

**DOI:** 10.3390/ijerph182413183

**Published:** 2021-12-14

**Authors:** Chiyoung Lee, Jee-Seon Yi

**Affiliations:** 1 School of Nursing & Health Studies, University of Washington Bothell, Bothell, WA 98011, USA; clee33@uw.edu; 2 Institute of Health Sciences, College of Nursing, Gyeongsang National University, Jinju 52727, Korea

**Keywords:** aged, Korea, latent class analysis, women, socioeconomic factors

## Abstract

Oldest-old women are known to live at the intersection of multiple socioeconomic disadvantages in South Korean society. This study classified oldest-old Korean women into several socioeconomically homogeneous classes based on various socioeconomic status (SES) risks and compared health characteristics among the identified classes. This cross-sectional study utilized the 2019 Korean Community Health Survey, including data from 11,053 women (≥80 years). Latent class analysis determined the number of underlying socioeconomic classes based on nine selected SES variables. Four distinct socioeconomic classes were identified: “Urban, living alone, recipient of NBLSS, moderate education, leisure activity” (Class 1), “Rural, traditional house, living with others, not financially deprived, low education, employed” (Class 2), “Urban, living with family, financially affluent, not employed, no barriers to healthcare” (Class 3), “Rural, traditional house, living alone, financially deprived, uneducated, employed, barriers to healthcare” (Class 4). Depressive symptoms, subjective stress, and the prevalence of sleep disorder and diabetes were higher in Class 1 compared to other classes. Health-related quality of life, perceived health, and self-rated oral health were the poorest in Class 4. Class 3 reported the best health status. Understanding the intersecting SES risk factors in this group can aid in developing targeted interventions.

## 1. Introduction

Socioeconomic status (SES) is a major determinant of health in late life. Especially in South Korea (hereafter “Korea”), with a rapidly aging population, there has been growing research interest in examining the influence of SES on the health of older adults [1]. The most frequently used proxy variable for the socioeconomic context in this age group is household income. Generally, older adults who have a lower income are reported to have a higher possibility of experiencing physical challenges, suffer from mental health disorders, and may encounter more functional impairments than their counterparts [1].

Some older adult studies that have examined the connection between income and health outcomes found no relationship [2,3], or associations in some groups only, for example, in women [4]. A possible reason for these conflicting results may be that income is mediated by a variety of other SES factors, and that single measures of SES are insufficient to capture SES in old age reliably and are not enough to explain health status variations fully [5]. For instance, area-based studies demonstrate that the socioeconomic conditions of localities also affect older adults’ health in addition to individual conditions [6,7]. Other socioeconomic issues such as employment, education, housing, segregation, and mobility can also make a difference in the health status of older adults [8]. This underscores the theory of intersectionality, which acknowledges that various socially constructed categories of differences interact to contribute to health disparities [9].

The above discussion is particularly relevant for oldest-old Korean women (≥80 years). They are a particularly marginalized population who have been exposed to decades of socioeconomic disadvantages due to Korea’s unique history [10]. Influenced by Confucian cultural values, these women constitute a larger low-SES portion of the population than older men in terms of income, occupational prestige, education level, or living standards [10]. Kim and Ruger reported more pronounced socioeconomic disparities in health for oldest-old women than men in their study [11] and argued that the overall inferior status of women in Korean society affects the gender differential in the impact of SES on health. In addition, women have been adversely affected by the socioeconomic support and healthcare system and have experienced widening disparities in access to healthcare services [12,13]. Further, with population aging, Korean society is now experiencing an increase in the number of oldest-old women living in rural areas without close residential propinquity with kin and relatives [12], all of which make these women live at the intersection of multiple socioeconomic disadvantages.

Still, taking into account the multidimensional aspects of socioeconomic disparities in health among oldest-old Korean women can be challenging for the researcher. Most of all, by including more than one SES indicator in a regression model—the most frequently used statistical analysis in design—we can violate the underlying assumptions of this analysis because of collinearity (i.e., high correlations between SES indicators). To overcome such problems, many approaches have been used to create composite indices, which use multiple SES indicators, to reflect a more holistic picture of SES, such as principal component analysis [14,15]. Despite being able to summarize multiple variables into one continuous variable, these indices are still unable to identify and describe patterns regarding the intersection of these variables.

A commonly used intersectional method for quantitative analyses is latent class analysis (LCA). LCA is a person-centered approach that divides a population into mutually exclusive and exhaustive groups based on observable indicator variables [16]. Importantly, LCA models allow for the characterization of SES into meaningful classifications, easily described by relevant SES indicators, and for estimating the effects of socioeconomic characteristics on outcomes of interest [17]. International studies have used this technique to estimate the burden of health problems attributable to different SES dimensions and identify individuals who could benefit from targeted interventions [17,18,19,20]. In particular, those identified to be in multiple disadvantaged SES have been shown to be at a greater risk of reporting health impairments than those in singly disadvantaged or privileged SES. However, to the best of our knowledge, no such effort has been made in Korea.

### Objective and Hypotheses

Accordingly, our project used Korean Community Health Survey (KCHS) data and LCA to identify distinct socioeconomic classes among oldest-old women in Korea and explore the socioeconomic contexts explaining this group’s essential health characteristics. The study hypotheses were as follows: (1) meaningful, distinct socioeconomic classes among older Korean women can be identified in the KCHS through LCA; (2) class membership is related to relevant health characteristics.

Importantly, our analytic method was informed by the theory of intersectionality. This theory helps researchers to be more explicit about why they include particular variables as important dimensions of disparity in research [21]. In the present study, we used a broad definition of socioeconomic disadvantage that reflects individuals’ access to material and social resources and their ability to participate in society [5]. This includes standard individual SES variables (e.g., income level, employment status, etc.) as well as the neighborhood socioeconomic context (e.g., barriers to accessing healthcare, convenience for leisure activities, etc.) as both are important health correlates [20,22,23] and are likely to intersect to affect the health of oldest-old women. Further, access to healthcare and leisure services have been regarded to strongly mirror one’s SES [5,24].

## 2. Materials and Methods

### 2.1. Data Source and Study Sample

This was a cross-sectional study based on 2019 KCHS data gathered by the Korea Centers for Disease Control and Prevention. Currently, in geriatric research in Korea [25,26], researchers classify older adults into three groups: the young-old (60–69), old-old (70–79), and oldest-old (≥80 years). Thus, our study’s population was limited to women 80 years of age or older. Detailed descriptions of the KCHS can be found on their website (https://chs.cdc.go.kr/chs/main.do, accessed date 3 July 2021). The KCHS comprises free public data that do not include personal information. However, we received institutional review board approval from the institution with which the corresponding author is affiliated (protocol number: GIRB-G21-X-0028). A total of 17,790 individuals age 80 or older participated in the KCHS survey in 2019. From this sample, we used only data from female participants (*n* = 11,524). Next, we removed missing data with respect to our variables of interest (*n* = 471). This yielded a sample of 11,053 participants for our study.

### 2.2. Selection and Manipulation of Variables

This study used answers to KCHS questionnaire items that were appropriate to the study purpose. Some SES variables were manipulated and categorized as LCA only handles categorical data. Finally, we chose candidates for health characteristic variables as factors potentially associated with the selected SES variables.

Socioeconomic variables. The SES variables include residential location (urban or rural), housing pattern (traditional house or apartment), living arrangement (living alone, living with a spouse, or living with family or others), income level (<1 million KRW, 1–2 million KRW, 2–3 million KRW, or ≥3 million KRW), current recipient of National Basic Livelihood Security System (NBLSS; yes or no), educational level (no formal education, elementary school, middle school, or high school or higher), employment status (yes or no), barriers to accessing healthcare (yes, no, or no need for health care), and leisure activity (yes or no). Residential location was classified as “urban” if the participant lived in a city (“*dong*” in Korean) in the administrative division or as “rural” if the respondent lived in a town (“*eup”*) or township (“*myeon”*). Barriers to accessing healthcare were assessed with a binary question: “Have you encountered any barriers when accessing the healthcare system in the past year for any reason?” In the 2019 KCHS survey, barriers to accessing healthcare included geographical barriers (e.g., transportation difficulties, distance to care, social isolation), financial constraints, limited healthcare facilities, or physician shortages. Leisure activity was answered as yes if the participants were able to enjoy leisure activity sufficiently in their socio-physical surroundings.

Health characteristics. Health-related variables include depressive symptoms (continuous), sleep disorder (yes or no), subjective stress (ordinal; 1 “rarely”, 2 “sometimes, 3 “often”, 4 “very much”), health-related quality of life (HRQoL; continuous), perceived health (ordinal; 1 “very poor” to 5 “very good”), self-rated oral health (ordinal; 1 “very poor” to 5 “very good”), diabetes (yes or no), and hypertension (yes or no). Depressive symptoms were assessed using the Patient Health Questionnaire (PHQ-9) using nine symptoms that make up the diagnosis of major depressive disorders, rating each item on a 0–3 scale based on whether the symptom bothered the respondent in the last two weeks [25]. The items were summed for a total score, with higher scores indicating higher levels of depressive symptomatology. The PHQ-9 is a well-established validated tool with high accuracy for screening, recommended in clinical practice [25]. Daily sleep duration of 5 h or less or more than 9 h was defined as a sleep disorder [26]. HRQoL was assessed by use of the EuroQoL EQ-5D Index [27]. The EQ-5D includes five dimensions (mobility, self-care, usual activities, pain/discomfort, anxiety/depression) evaluated on a scale of 1 (no problems) to 3 (severe problems), which was reverse-coded to reflect high score as higher HRQoL in the present study. The average score of five dimensions that contribute to the scale was calculated in order to determine the HRQoL score.

### 2.3. Statistical Analysis

LCA was employed to determine the number of underlying socioeconomic classes in the total sample based on nine selected SES variables. LCA estimates the posterior probabilities of class membership (the probability that an individual belongs to a particular class) using two parameters termed class probabilities and item probabilities [16]; while the former estimates the percentage of participants who belong to each class, the latter estimates the probability with which each class member endorses an item. The optimal class solution was selected based on various model fit statistics and theoretical interpretability. The fit statistics reported in the current study were the Lo–Mendell–Rubin’s adjusted likelihood ratio test (LRT), entropy measures, Akaike information criterion (AIC), Bayesian information criterion (BIC), and sample-size adjusted BIC (SSABIC). The Lo–Mendell–Rubin’s LRT statistic was used to compare models with different numbers of latent classes; a non-significant value (*p* > 0.05) suggests that the model with one fewer class should be accepted. Entropy is a standardized measure of how accurately participants are classified and ranges from 0 to 1, with greater indicating better classification accuracy. The AIC, BIC, and SSABIC are goodness-of-fit measures used to compare competing models; lower values reflect better fitting models.

Once the classes were identified, a name was chosen for each class that best described its characteristics and differentiated it from other classes. Group characteristics were examined using the chi-squared test to determine if classes derived from LCA differed significantly. Next, bivariate analyses were used to assess the associations between class membership and health characteristics: ANOVA was used for continuous variables and the chi-squared test for categorical variables. All analyses were performed using Mplus 7.4 software (Muthen and Muthen, Los Angeles, CA, USA).

## 3. Results

The results were “unweighted,” as we could not incorporate a complex survey design (e.g., sampling weights, strata, and primary sampling units) into the analyses due to the current lack of LCA methodologies accommodating complex design features. Hereafter, we refer to unweighted prevalence rates (or unweighted means) directly as prevalence rates (or means) and provide further discussion in the limitations section.

### 3.1. Fit Statistics for 1–6 Latent Classes

Table 1 illustrates the latent class model fit indices. The LMR test did not indicate a significant improvement of fit from the 5-class to 6-class model at the 0.05 level; thus, we considered statistical evidence and theoretical interpretability only for models with 1–5 classes. We did not consider the 5-class model, as its entropy was below the acceptable level (0.786). In addition, although the entropy was superior in models with fewer classes, the fit indices—including the AIC, BIC, SABIC, and log-likelihood—had markedly lower values for the 4-class solution than for the 2-class and 3-class solutions. Lastly, compared to the 3-class solution, the 4-class solution provided additional meaningful information that further distinguished the classes and increased their conceptual interpretability. Considering the overall patterns, we determined the 4-class model to be optimal.

### 3.2. Distribution of SES Risks in the 4-Class Model

The distribution of SES risks in the 4-class model is described in Table 2. The SES risk variables significantly differed among the 4 classes (all *p* < 0.001). Class 1 and 3 included a large proportion of people living in urban areas (88.9% and 90.3%, respectively), whereas a large proportion of people in Class 2 and 4 were living in rural areas (94.1% and 90.4%, respectively). Interestingly, nearly all people in Class 2 and 4 reported living in a traditional house. Class 1 and 4 had the highest rates of people living alone (63.6% and 78.1%, respectively); in contrast, Class 2 and 4 had the highest rates of people living with family (76.1% and 90.1%, respectively). In addition, approximately 20% of the people in Class 1 and 2 reported living with a spouse.

In terms of income level, Class 1 included more than half the proportion of people in the poorest income category (59.3%). Class 2 reported individuals living in every income category except the poorest. Class 3 included the largest proportion of people in the highest income category (67.2%). Almost all people classified in Class 4 reported belonging to the poorest income category (95.2%). Older adults in Class 1 were more likely to be recipients of NBLSS than those in other classes (19.3%). In terms of educational level, Class 4 had the largest proportion of people who had no formal education (81.7%), whereas Class 1 had the least (37.0%). Class 2 also constituted high rates of individuals with no formal education (76.0%). Compared to other classes, Class 1 and 3 had a relatively higher proportion of people whose educational level was high school or higher (12.7% and 11.6%). In addition, a relatively high number of employed people were represented in Class 2 (22.8%) and 4 (21.0%), whereas hardly any participants in Class 1 and 3 were currently employed (5.6% and 2.0%, respectively).

Although older adults overall replied that they did not experience barriers to accessing healthcare (88.8% of the total), individuals in Class 4 were more likely to experience barriers than other classes (10.8%). Lastly, older adults in Class 1 and 3 were more likely to enjoy leisure activity (10.1% and 8.1%, respectively) than those in Class 2 and 4 (2.6% and 2.3%, respectively). Table 3 provides the names and short descriptions of the latent classes from the 4-class solution.

### 3.3. Inter-Class Health Characteristic Differences

Significant differences in health characteristics were noted among classes (Table 4). The four classes differed significantly in depressive symptoms, subjective stress, the prevalence of sleep disorder, HRQoL, subjective health, self-rated oral health, and the prevalence of diabetes (*p* < 0.001). Specifically, Class 1 had the highest score of depressive symptoms (13.05 ± 4.35), while Class 2 had the lowest (12.26 ± 3.94). Similarly, Class 1 constituted the largest proportion of people who were experiencing a sleep disorder (36.5%), whereas Class 2 constituted the smallest (28.5%). Class 1 comprised the highest proportion of people who reported higher levels of stress (i.e., “often”, “very much”), but Class 3 reported the lowest. Notably, similar to Class 1, Class 4 showed relatively higher scores for depressive symptoms and had a larger proportion of individuals with higher levels of stress and a sleep disorder compared to the other classes.

HRQoL was equally high in Class 1 and 3 compared to the other two classes, and it was lowest in Class 4. Class 3 had the highest score for perceived health status (2.47 ± 0.93), whereas Class 4 had the lowest (2.26 ± 0.90). Self-reported scores for oral health were highest in Class 1 (2.26 ± 0.99) and lowest in Class 4 (2.13 ± 0.92). Lastly, Class 1 and 3 reported a higher prevalence of diabetes than the other classes. However, no significant differences were observed in the prevalence of hypertension among classes.

## 4. Discussion

In the present study, four distinct socioeconomic classes among Korean oldest-old women were identified with several satisfactory fit indices using LCA—“Urban, living alone, recipient of NBLSS, moderate education, leisure activity” (Class 1), “Rural, traditional house, living with others, not financially deprived, low education, employed” (Class 2), “Urban, living with family, financially affluent, not employed, no barriers to healthcare” (Class 3), “Rural, traditional house, living alone, financially deprived, uneducated, employed, barriers to healthcare” (Class 4)—which supported our hypothesis. Additionally, in accordance with our hypothesis, class membership was significantly associated with relevant health characteristics.

First, depressive symptoms, the prevalence of sleep disorder, subjective stress, and the prevalence of diabetes were higher in Class 1 (“Urban, living alone, recipient of NBLSS, moderate education, leisure activity”) compared to the other classes. It is worthwhile to closely examine the observed intersection of Class 1′s family type (i.e., living alone), Class 1′s health characteristics, and other influential socioeconomic factors. Although the role of living arrangements in older adults’ mental health differs across societies and cultures, a commonly held view is that the traditional Korean extended family could mitigate mental health risks among older adults by providing economic and emotional support [28]. In several Korean studies [29,30,31], living alone has been viewed as an undesirable state and a risk factor for depression and stress; this is particularly relevant for older women living in cities, where family ties are weaker and older women experience difficulties making friends with dependable peers [31]. Further, living alone is associated with financial, social, lifestyle, and environmental factors that are likely to influence nutrition behaviors [32], which in turn may lead to chronic diseases, such as type 2 diabetes [33]. Our findings about Class 1 strengthen the existing evidence in this domain.

Notably, a greater number of NBLSS recipients were categorized in Class 1 compared to the other classes. Ju et al. [34] argued that elderly Korean welfare recipients often experience not only economic difficulties but also emotional anxiety, as they tend to neglect their health to maintain a basic livelihood on a strict budget and often do not have family members to support them; even if they do have a family member to support them, that individual is often not capable of providing them with sufficient means of livelihood, which exacerbates their psychological instability and distress. Interestingly, in Ju et al.’s [34] qualitative study of Korean elderly welfare recipients’ daily life experiences, some of the major themes were “daily lives enduring physical and mental sufferings alone and mismatches of social welfare services” and “mind and body withered by economic hardship in winter”, which may partially reflect the characteristics of Class 1 identified in the present study.

It was also noticeable that Class 1 comprised a large proportion of oldest-old women residing in urban areas. It was not feasible to directly compare our results with previous studies that adopted an intersectional approach [17,18,19,20] as they hardly incorporated the geographic attributes. Further, the literature on geographic associations with depression is inconclusive. Some studies showed that older adults living in cities have poorer mental health due to environmental factors, including excessive exposure to artificial lighting and nighttime crime, than rural residents [35,36,37]. Particularly, Min and Min [37] argued that in urban Korean cities, artificial nighttime lighting has been the fastest-growing environmental pollutant that causes sleep disturbances and is significantly associated with prescription of hypnotic drugs for older adults. However, others have cited lower depression rates in urban areas [37,38,39]. In a similar fashion, our finding on Class 1 partially extends former research reporting a higher prevalence of diabetes among older adults living in urban areas than in those living in rural areas [40,41].

HRQoL, perceived health, and self-rated oral health were the poorest in Class 4 (“Rural, traditional house, living alone, financially deprived, uneducated, employed, barriers to healthcare”). Indeed, HRQoL reflects an individual’s subjective satisfaction and well-being [42] and is closely related to perceived health [43] and oral health status [43]. Thus, it is interesting to see how these three levels of health are combined with other socioeconomic factors to produce Class 4′s characteristics. In fact, this result accords with past findings. For instance, Yi [43] showed that oldest-old women living in rural/traditional settings in Korea have lower HRQoL, especially when experiencing low levels of subjective health and unmet healthcare needs. Additionally, Yang et al. [44] report that, especially in the oldest-old, a lack of knowledge about oral health, substantial medical expenses, and avoidance of medical treatment contribute to poor oral health, ultimately lowering HRQoL.

Class 4 indeed was the most socioeconomically vulnerable group, living at the intersection of multiple disadvantages in terms of living conditions, income, education, and access to healthcare. Importantly, studies show that these disadvantages reciprocally influence each other to produce negative outcomes for older women [10,12,45]. Although not perfectly analogous, Choi and Kim’s [45] study demonstrated that unmet healthcare needs are higher among older Korean women living in rural areas (e.g., transportation inconvenience was a major healthcare barrier), living alone, having lower educational attainment, and depending on a basic livelihood subsidy, all of which influence their poor subjective health. Given that more than 50% of the participants were categorized in Class 4, increased efforts should be directed to improving this group’s health.

Meanwhile, it is apparent that Class 4 (which had the lowest HRQoL score) had a greater proportion of employed members compared to the other classes. This contrasts with previous studies’ findings that employment is an important factor affecting QoL for older adults, as it is a means of interacting with many individuals [46,47]. Older adults working in traditional rural Korean communities are often engaged in community-based farming and leisure activities and report a higher QoL [46]. However, it can be argued that for the participants in Class 4, a job may be a means of earning a livelihood given their disadvantaged conditions. Min [48] also found that QoL among Korean women is not affected by job retention or employment but is rather strongly affected by living alone.

Class 3 (“Urban, living with family, financially affluent, not employed, no barriers to healthcare”) had the best health status overall. The diverse socioeconomic context associated with Class 3 may thus help in creating strategies for health promotion and decreasing health disparities among older Korean women. For instance, policies focused on strengthening the supply of primary care services or on promoting an integrated network of services would contribute to improving access and reducing the financial burden. Further, a needs assessment may be helpful to explore specific reasons for barriers to accessing services. In addition, the use of social networks that provide family-like support to older women should be activated. Meanwhile, despite their declining health, HRQoL was significantly higher in Class 1 than in the other classes. This may be because Class 1 had a higher rate of engagement in leisure activities compared to the other classes. The opposite trend found for Class 4 strengthens our argument. Indeed, one qualitative study also showed that oldest-old women experience high QoL by finding purpose in life through participation in daily leisure activities, volunteer programs, and spiritual gatherings, regardless of their deteriorating health [49]. Therefore, future research should closely evaluate the role of leisure activities among older women.

### Limitations and Strengths

This study has several limitations. First, we could not consider sample design features (i.e., sample weighting, clustering, and stratification) used in complex surveys due to the lack of established methods for LCA models. For instance, the LRT is only available for unweighted estimates [50]. Accordingly, our sample should not be considered truly representative of community-dwelling oldest-old Korean women in 2019. Nonetheless, researchers should note that our results emerged from analysis of an extensive national survey with a diverse group of participants and with generalizability possibly superior to smaller or convenience samples. At the same time, one should note that, if data are collected under a complex sampling design but simple random sampling is assumed in the analysis, then model parameter estimates can be biased, and the measure of variances such as standard error can be underestimated [50]. Some caution is thus needed in drawing any conclusions based on these estimates. Second, the results were based on self-report data, which may have compromised the validity of answers to sensitive questions. Third, our results are based on a cross-sectional survey; therefore, the analyses cannot establish causal relationships. Further, selection or reverse causation (health to SES) cannot be ruled out. For instance, impaired health and functioning among older adults may reduce income generation through expenditures for healthcare [5]. Future studies should thus adopt measures (e.g., using wealth as a proxy for economic resources instead of income) or analyses that are less affected by reverse causation (e.g., longitudinal analyses). Fourth, one should keep in mind that the study of the oldest-old can be affected by survivorship bias (i.e., our participants who survived for a long time would be healthier), and this may limit the external validity of the findings observed. In addition, failing to account for survivorship bias may result in an underestimation of socioeconomic differences in health. Fifth, our use of secondary data for analysis limited our choice of variables to those available in the dataset. For instance, apart from hypertension and diabetes, other chronic conditions such as cancer and arthritis, which were also closely related to SES characteristics among older adults, were not included in the 2019 KCHS data. Lastly, the original variables included in the secondary data potentially lacked depth because they were operationally defined by a single survey item or a subset of test items. This may have influenced their estimated strengths as independent variables for the LCA analysis.

Several of the LCA’s limitations should also be noted. First, even though LCA facilitates identifying associations among baseline SES variables that may differ in strength across classes, it does not accurately reveal the specific variables driving these associations [51]. Second, even though grouping based on latent class facilitates data presentation and interpretation, the participants do not actually belong to a single class. The class membership of each participant is assigned based on the highest probability of belonging to one of the latent classes [51]. In other words, some participants might have similar probabilities of belonging to multiple classes (i.e., probabilities of 0.49, 0.48, 0.01, and 0.02 for Classes 1, 2, 3, and 4, respectively); however, the class membership is assigned based on the highest probability. Thus, it is vital to analyze participants for whom the highest probability of belonging to a single class is poor (<0.7) and further describe such participants [52].

Despite these limitations, our study has important strengths. Above all, several intersecting SES risk factors identified in this study could be vulnerability indicators, identifying individuals who require more targeted screening, early detection, or focused health attention. In addition, our results can be used as a basis for eliminating health disparities among oldest-old Korean women.

## 5. Conclusions

Informed by intersectionality, LCA allowed us to classify oldest-old Korean women into several socioeconomically homogeneous classes based on various SES risks and to compare health characteristics among the identified classes. Consequently, we identified four distinct socioeconomic classes, and class membership was significantly associated with relevant health characteristics. Depressive symptoms, the prevalence of sleep disorder, subjective stress, and the prevalence of diabetes were higher in Class 1 (“Urban, living alone, recipient of NBLSS, moderate education, leisure activity”) compared to the other classes. HRQoL, perceived health, and self-rated oral health were the poorest in Class 4 (“Rural, traditional house, living alone, financially deprived, uneducated, employed, barriers to healthcare”). However, Class 3 (“Urban, living with family, financially affluent, not employed, no barriers to healthcare”) reported the best overall health.

Our study has several implications for future research, clinical practice, and policy. First, our results shows that LCA can be a fruitful way of studying socioeconomic patterning among older women. Second, segmenting populations into specific classes based on SES risks may improve the scope, utilization, and efficacy of interventions. Third, various health factors associated with class membership may inform tailored interventions. Ultimately, the key challenge for public health professionals is to design tailored health policies that consider socioeconomic variability among oldest-old Korean women.

## Figures and Tables

**Table 1:** Fit statistics for 1–6 latent classes (*N* = 11,053).

No. of Classes	Number of Each Class	LRT	Entropy	AIC	BIC	SSABIC	Log-Likelihood
1	C1 = 11,053	NA	1.000	119,080.43	119,190.09	119,142.42	−59,525.22
2	C1 = 7368, C2 = 3685	2 vs. 1 Value = −59,525.22, *p* < 0.001	0.847	111,335.27	111,561.89	111,463.38	−55,636.63
3	C1 = 2979, C2 = 6562, C3 = 1512	3 vs. 2 Value = −55,636.63, *p* < 0.001	0.832	108,685.07	109,028.66	108,879.30	−54,295.53
**4**	**C1 = 1690, C2 = 2045, C3 = 1468, C4 = 5850**	**4 vs. 3** **Value = −54,295.61, *p* < 0.001**	**0.820**	**107,371.17**	**107,831.73**	**107,631.520**	**−53,622.58**
5	C1 = 1593, C2 = 1353, C3 = 5207, C4 = 1199, C5 = 1701	5 vs. 4 Value = −53,622.58, *p* < 0.001	0.786	106,638.25	107,215.78	106,964.72	−53,240.13
6	C1 = 1300, C2 = 1593, C3 = 1210, C4 = 1714, C5 = 563, C6 = 4673	6 vs. 5 Value = −53,240.13, *p* = 0.913	0.785	106,204.54	106,899.03	106,597.14	−53,007.27

Note: LRT, Lo–Mendell–Rubin’s adjusted likelihood ratio test; AIC, Akaike information criteria; BIC, Bayesian information criteria; aBIC, adjusted Bayesian information criteria; SSABIC, sample-size adjusted BIC. The 4-class model (written in bold) was selected as the optimal model for interpretation and additional analysis.

**Table 2:** Distribution of SES risks in the 4-class model.

SES Variables	Total (*N* = 11,053)	Comparisons among Latent Classes				*p*-Value *
		Class 1 (*n* = 1690)	Class 2 (*n* = 2045)	Class 3 (*n* = 1468)	Class 4 (*n* = 5850)	
Residential location						<0.001
Rural	7540 (68.2)	187 (11.1)	1924 (94.1)	143 (9.7)	5286 (90.4)	
Urban	3513 (31.8)	1503 (88.9)	121 (5.9)	1325 (90.3)	564(9.6)	
Housing pattern						<0.001
Traditional house	9096 (82.3)	651 (38.5)	2041 (99.8)	653 (44.5)	5751 (98.3)	
Apartment	1957 (17.7)	1039 (61.5)	4 (0.2)	815 (55.5)	99 (1.7)	
Living arrangement						<0.001
Living alone	5681 (51.4)	1074 (63.6)	14 (0.7)	26 (1.8)	4567 (78.1)	
Living with a spouse	1860 (16.8)	382 (22.6)	475 (23.2)	120 (8.2)	883 (15.1)	
Living with a family	3512 (31.8)	234 (13.8)	1556 (76.1)	1322 (90.1)	400 (6.8)	
Income level						<0.001
<1 million KRW	6570 (59.4)	1003 (59.3)	0 (0.0)	0 (0.0)	5567 (95.2)	
1–2 million KRW	1892 (17.1)	606 (35.9)	871 (42.6)	132 (9.0)	283 (4.8)	
2–3 million KRW	921 (8.3)	81 (4.8)	490 (24.0)	350 (23.8)	0 (0.0)	
≥3 million KRW	1670 (15.1)	0 (0.0)	684 (33.4)	986 (67.2)	0 (0.0)	
Current recipient of NBLSS						<0.001
Yes	961 (8.7)	326 (19.3)	62 (3.0)	1 (0.1)	572 (9.8)	
No	10,092 (91.3)	1364 (80.7)	1983 (97.0)	1467 (99.9)	5278 (90.2)	
Educational level						<0.001
No formal education	7611 (68.9)	626 (37.0)	1555 (76.0)	650 (44.3)	4780 (81.7)	
Elementary school	2584 (23.4)	677 (40.1)	442 (21.6)	505 (34.4)	960 (16.4)	
Middle school	427 (3.9)	173 (10.2)	27 (1.3)	143 (9.7)	84 (1.4)	
High school or higher	431 (3.9)	214 (12.7)	21 (1.0)	170 (11.6)	26 (0.4)	
Employment status						<0.001
No	9233 (83.5)	1595 (94.4)	1578 (77.2)	1438 (98.0)	4622 (79.0)	
Yes	1820 (16.5)	95 (5.6)	467 (22.8)	30 (2.0)	1228 (21.0)	
Barriers to accessing healthcare						<0.001
Yes	926 (8.4)	103 (6.1)	148 (7.2)	44 (3.0)	631 (10.8)	
No	9816 (88.8)	1556 (92.1)	1842 (90.1)	1391 (94.8)	5027 (85.9)	
No need for health care	311 (2.8)	31 (1.8)	55 (2.7)	33 (2.2)	192 (3.3)	
Leisure activity						<0.001
No	10,576 (95.7)	1520 (89.9)	1992 (97.4)	1349 (91.9)	5715 (97.7)	
Yes	477 (4.3)	170 (10.1)	53 (2.6)	119 (8.1)	135 (2.3)	

Note: NBLSS = National Basic Livelihood Security System. * *p*-value for chi-squared test.

**Table 3:** Description of latent classes from 4-class model solution for participants.

Class	Description
**Class 1** “Urban, living alone, recipient of NBLSS, moderate education, leisure activity”	Residing in an urban area Mostly living in an apartment About 64.0% living alone, 23.0% living with a spouse Moderate financial deprivation About 19.0% are recipients of NBLSS Medium level of education Low current employment Higher participation in leisure activity than other classes
**Class 2** “Rural, traditional house, living with others, not financially deprived, low education, employed”	Residing in a rural area Living in a traditional house Living with a spouse or family Not financially deprived Low level of education Moderate current employment Hardly participates in leisure activity
**Class 3** “Urban, living with family, financially affluent, not employed, no barriers to healthcare”	Residing in an urban area About 90.1% living with family Financially affluent Not a recipient of NBLSS Medium level of education Low level of education No current employment No barriers to accessing healthcare Relatively high participation in leisure activity than other classes
**Class 4** “Rural, traditional house, living alone, financially deprived, uneducated, employed, barriers to healthcare”	Residing in a rural area Living in a traditional house About 78.1% living alone Financially deprived About 10.0% are recipients of NBLSS No formal education Moderate current employment Experiences more barriers to accessing healthcare than other classes Hardly any participation in leisure activity

Note: NBLSS = National Basic Livelihood Security System.

**Table 4:** Inter-class health characteristic differences.

Health-Related Variables	Total (*N* = 11,053)	Comparisons among Latent Classes (Mean ± SD, *n* (%))				*p*-Value *
		Class 1 (*n* = 1690)	Class 2 (*n* = 2045)	Class 3 (*n* = 1468)	Class 4 (*n* = 5850)	
Depressive symptoms	12.70 ± 4.26	13.05 ± 4.35	12.26 ± 3.94	12.49 ± 4.20	12.81 ± 4.35	<0.001
Sleep disorder						<0.001
Yes	3648 (33.0)	617 (36.5)	584 (28.5)	420 (28.6)	2027 (34.6)	
No	7405 (67.0)	1073 (63.5)	1461 (71.5)	1048 (71.4)	3823 (65.4)	
Subjective stress						<0.001
Rarely	5120 (46.3)	710 (42.0)	977 (47.8)	702 (47.8)	2731 (46.7)	
Sometimes	3992 (36.1)	659 (39.0)	750 (36.7)	566 (38.6)	2016 (34.5)	
Often	1643 (14.9)	271 (16.0)	273 (13.3)	180 (12.3)	918 (15.7)	
Very much	298 (2.7)	50 (3.0)	45 (2.2)	20 (1.4)	183 (3.1)	
HRQoL	2.39 ± 0.42	2.417 ± 0.43	2.40 ± 0.42	2.416 ± 0.43	2.38 ± 0.41	<0.001
Perceived health	2.26 ± 0.91	2.36 ± 0.92	2.34 ± 0.91	2.47 ± 0.93	2.26 ± 0.90	<0.001
Self-rated oral health	2.18 ± 0.92	2.26 ± 0.99	2.18 ± 0.91	2.25 ± 0.91	2.13 ± 0.92	<0.001
Diabetes						<0.001
Yes	2216 (20.0)	437 (25.9)	356 (17.4)	320 (21.8)	1103 (18.9)	
No	8837 (80.0)	1253 (74.1)	1689 (82.6)	1148 (78.2)	4747 (81.1)	
Hypertension						0.245
Yes	7147 (64.7)	1128 (66.7)	1304 (63.8)	942 (64.2)	3773 (64.5)	
No	3906 (35.3)	562 (33.3)	741 (36.2)	526 (35.8)	2077 (35.5)	

Note: Daily sleep duration of 5 h or less or more than 9 h was defined as a sleep disorder. HRQoL = health-related quality of life. SD = standard deviation. * *p*-value for ANOVA or chi-squared test.

## Data Availability

For users and researchers throughout the world, microdata (in the form of SAS files) and analytic guidelines can be downloaded from the KCHS website (http://KCHS.cdc.go.kr/, accessed date 3 July 2021) in Korean.
